# Screening and Validation: AI-Aided Discovery of Dipeptidyl Peptidase-4 Inhibitory Peptides from Hydrolyzed Rice Proteins

**DOI:** 10.3390/foods14111916

**Published:** 2025-05-28

**Authors:** Cheng Cheng, Huizi Cui, Xiangyu Yu, Wannan Li

**Affiliations:** Key Laboratory for Molecular Enzymology and Engineering of Ministry of Education, School of Life Sciences, Jilin University, Changchun 130012, China; chengcheng2222@mails.jlu.edu.cn (C.C.); hzcui23@mails.jlu.edu.cn (H.C.); yuxy1321@mails.jlu.edu.cn (X.Y.)

**Keywords:** dipeptidyl peptidase-4, rice protein hydrolysates, molecular docking, molecular dynamics simulation, virtual screening

## Abstract

Dipeptidyl peptidase-4 (DPP-4) inhibitors play a critical role in the management of type 2 diabetes; however, some synthetic drugs may cause adverse effects. Natural peptides derived from rice offer a promising alternative due to their favorable biocompatibility and development potential. In this study, an AI-assisted virtual screening pipeline integrating machine learning, molecular docking, and molecular dynamics (MD) simulations was established to identify and evaluate rice-derived DPP-4 inhibitory peptides. A random forest classification model achieved 85.37% accuracy in predicting inhibitory activity. Peptides generated by simulated enzymatic hydrolysis were screened based on machine learning and docking scores, and four proline-rich peptides (PPPPPPPPA, PPPSPPPV, PPPPPY, and CPPPPAAY) were selected for MD analysis. The simulation results showed that PPPSPPPV formed a stable complex with the DPP-4 catalytic triad (Ser592–Asp670–His702) through electrostatic and hydrophobic interactions, with low structural fluctuation (RMSF < 1.75 Å). In vitro assays revealed that PPPPPY exhibited the strongest DPP-4 inhibitory activity (IC_50_ = 153.2 ± 5.7 μM), followed by PPPPPPPPA (177.0 ± 6.0 μM) and PPPSPPPV (216.3 ± 4.5 μM). This study presents an efficient approach combining virtual screening and experimental validation, offering a structural and mechanistic foundation for the development of natural DPP-4 inhibitory peptides as candidates for functional foods or adjunct diabetes therapies.

## 1. Introduction

Diabetes mellitus, particularly type 2 diabetes, has emerged as a global health crisis, with its prevalence escalating due to lifestyle changes and aging populations [1]. There were 537 million people living with diabetes worldwide in 2021, and future projections suggest that by 2045, the number of people with diabetes will have increased by 46%, with the greatest absolute growth in terms of the number of persons with diabetes occurring in middle-income countries, such as China [2]. In China, the marked increase in the diabetes prevalence is mostly attributed to type 2 diabetes [3], and the major risk factors that have contributed to the dramatic rise in type 2 diabetes in China include obesity and diet [4]. So, when choosing glucose-lowering medications for people with type 2 diabetes and overweight or obesity, priority should be given to medications with a beneficial effect on weight according to the American Diabetes Association (ADA). Dipeptidyl peptidase-4 (DPP-4) inhibitors demonstrate clinical advantages, including weight neutrality, mild hypoglycemic effects, and sustained glycemic control, positioning them as a safer and more mechanism-driven therapeutic option compared to conventional antidiabetic drugs [5,6].

DPP-4 cleaves dipeptides from the N-terminal of peptides containing Pro or Ala residues. Glucose-dependent insulinotropic polypeptide (GIP) and glucagon-like peptide-1 (GLP-1) are endogenous substrates of DPP-4, and their proteolytic inactivation by DPP-4 impairs insulin secretion and glucose homeostasis [7]. DPP-4 inhibitors, including sitagliptin and saxagliptin, have gained clinical prominence by prolonging GLP-1 activity [8,9]. Intriguingly, computational models suggest a robust molecular interaction between the SARS-CoV-2 S1 subunit and DPP-4 [10], while COVID-19 may share infection patterns with SARS-CoV and MERS-CoV [11]. Clinical investigations during the COVID-19 pandemic indicated that infected individuals with diabetes are prone to more complex clinical manifestations. DPP-4 inhibitors may serve as immunomodulatory agents in COVID-19, potentially reducing the mortality risks in patients with diabetes or other comorbid conditions by mitigating respiratory inflammation [12].

Existing synthetic inhibitors may exhibit adverse effects [13], driving the search for novel, cost-effective, and biocompatible alternatives [14]. Bioactive peptides from natural sources may become the source of DPP-4 inhibitors [15]. Among these plant proteins, some have attracted attention due to their potential safety, structural diversity, and functional specificity [16]. Peptides from soybean, rice and wheat protein hydrolysates demonstrate that peptides produced by hydrolysis of natural plant proteins may have DPP-4 inhibitory activity [17,18,19]. Among these, rice is a staple food throughout Asia and has abundant protein reserves. The protein content in rice is 5–7% by weight, consisting of glutelin (80%), globulin (4–15%), prolamin (2–8%) and albumin (1–5%) [20]. Research has found that the α-amylase and β-glucosidase inhibitory activities of albumin and glutelin hydrolysates from rice bran protein are comparable in magnitude to those of the standard anti-diabetic drug acarbose [19]. Other studies have demonstrated that oral proteolytic hydrolysates prepared from rice effectively reduce the glycemic response by increasing the GLP-1 and insulin levels [21]. However, traditional screening methods for identifying DPP-4 inhibitory peptides from rice remain labor-intensive and inefficient. Machine learning (ML) and virtual hydrolysis offer transformative tools to accelerate peptide discovery and visualize the sequence of polypeptides [22,23,24]. Peptide property analysis in silico enables rapid prediction of its bioactivity and physicochemical properties, while molecular docking and dynamics simulations provide atomistic insights into peptide–enzyme interaction and conformational change [25].

Based on this, the primary objectives of this study were to identify natural and safe therapeutic agents for diabetes by elucidating the residue basis and conformational changes underlying the interactions between DPP-4 and its inhibitory peptides, as well as to present an integrated computational pipeline to screen and identify DPP-4 inhibitory peptides. The secondary objectives included exploring potential adjunctive therapies for COVID-19 and investigating the comprehensive value of plant protein resources.

This study identified the rice-derived peptide PPPSPPPV as a potent DPP-4 inhibitor through integrated machine learning, docking, and molecular dynamics (MD) simulations. PPPSPPPV binds with DPP-4 via the Glu205 salt bridge (ΔTOTAL = −29.22 kcal/mol) and Phe319/Tyr628 π-stacking, suppressing the catalytic triad fluctuations (RMSF < 1.75 Å) through α/3_10_-helix stabilization. The AI-enhanced pipeline establishes a rapid, precise strategy for discovering plant-derived therapeutics with optimized safety and mechanistic synergy.

## 2. Materials and Methods

### 2.1. Research Questions

The sequences of potential DPP-4 inhibitory peptide fragments; whether the screened inhibitory peptides possess DPP-4 inhibitory capability at the molecular level; whether their inhibitory efficacy can exceed that of the positive controls (IPI); and whether the physicochemical properties of the peptides—including the molecular weight, isoelectric point, hydrophobicity, net hydrogen and net charge—are correlated with their docking scores against DPP-4.

### 2.2. Machine Learning Model Training

#### 2.2.1. Dataset Construction

To construct a robust machine learning dataset, we curated 566 experimentally validated DPP-4 inhibitory peptides (positive samples) from the peer-reviewed literature (2003–2024) and publicly available databases. High-confidence negative samples were produced via random generation and selected as peptides with confirmed inability to bind DPP-4, such as those enriched in polar residues (Arg, Tyr, Glu) that induce charge repulsion at the hydrophobic catalytic site [26]. The literature came from PubMed (a free library of biomedical and life science documents), and the databases included PubChem (a chemical information website), ChEMBL (a bioactive molecules database of drug properties), and BindingDB (the first publicly available molecular recognition database). Although potential DPP-4 inhibitory activity may be present in some negative samples, it did not significantly affect the results [27]. This formed the dataset for the subsequent machine learning model.

#### 2.2.2. Descriptor Construction

The Morgan fingerprint (formally known as extended-connectivity fingerprints, ECFPs) is a circular topological descriptor widely used in cheminformatics for molecular structure representation and similarity analysis. It encodes the atom environments within a specified bond radius, enabling machine learning models to capture critical structural motifs [28]. In this study, we generated Morgan fingerprints using the RDKit cheminformatics toolkit, with the parameters set to a radius of 3 (capturing 3-bond neighborhoods), Bits = 1024, and $n_{Bits}=2048$ (defining the fingerprint bit vector length). The parameter search strategy was a grid search, with the cross-validation folds set to five-fold cross-validation.

While the theoretical diversity of Morgan fingerprints scales as $2^{n_{Bits}}$, the actual number of unique fingerprints in practice is constrained by the molecular structural diversity within the dataset. For example, our curated peptide dataset (N=566) yielded only 494 unique fingerprints (Equation (1)), reflecting limited sequence–structure variability.

(1) $${\;\;\;\;E}_{unique}\;\approx n_{Bits}\cdot \left(1-e^{-\frac{N}{n_{Bits}}}\right)\;\;\;\;\;\;\;\;(N\ll n_{Bits})$$

#### 2.2.3. Machine Learning Model Construction

The RF model, an ensemble method that aggregates predictions from multiple decision trees, was tuned for five key parameters: the number of trees (n_estimators: 50, 100, 200), splitting criterion (criterion: “gini” and “entropy”), maximum depth of the trees (max_depth: none, 5, 10, 15), minimum samples per leaf (min_samples_leaf: 1–10), and number of features considered at each split (max_features: “log2”, “auto”, “sqrt”). By constructing multiple decision trees from random subsets of the data, RF enhances the model robustness and predictive performance.

#### 2.2.4. Model Evaluation

Standard quantitative metrics, including the sensitivity (SE), specificity (SP), Matthews correlation coefficient (MCC), accuracy (ACC), precision (P), F1 score (F1), balanced accuracy (BA), and area under the ROC curve (AUC), as defined by Equations (2)–(7), were used to assess the RF model. The model performance was quantified using composite graphs and double-hundred confusion matrices.

(2) $$SE=\frac{TP}{TP+FN}$$

(3) $$SP=\frac{TN}{TN+FP}$$

(4) $$ACC=\frac{TP+TN}{TP+TN+FP+FN}$$

(5) $$P=\frac{TP}{TP+FP}$$

(6) $$F1=2\frac{PR}{P+R}$$

(7) $$\;BA=\frac{SE+SP}{2}$$

### 2.3. Data Acquisition and Protein Hydrolysis

Rice protein sequences were retrieved from the NCBI protein database (https://www.ncbi.nlm.nih.gov/). We selected glutelin and prolamin, which accounted for 80% and 2–8% of rice protein (Table S1), and hydrolysis in silico was performed using ExPASy-Peptide Cutter (https://web.expasy.org/peptide_cutter/, accessed on 12 November 2024) to simulate cleavage by single enzymes [Trypsin, Pepsin (PH > 2), Proteinase K and Chymotrypsin-high specificity (C-term to [FYW], not before P)] and enzyme combinations [Chymotrypsin-high specificity (C-term to [FYW], not before P) + Proteinase K and Trypsin + Pepsin (PH > 2)] under human digestive conditions. The hydrolysis parameters (pH, temperature, enzyme-to-substrate ratio) were set according to the website [29]. A total of 638 non-redundant peptides were generated after removing the duplicate sequences across all the enzymatic treatments (Table S2).

### 2.4. Virtual Analysis and Prediction

All the peptide segments in the dataset were analyzed for their physical and chemical properties and predicted for activity. ToxinPred2.0-Designing and prediction of toxic Peptides (https://webs.iiitd.edu.in/raghava/toxinpred/, accessed on 22 November 2024) was used to predict the physical and chemical properties [net charge, hydropathicity, hydrophilicity, hydrophobicity, molecular weight, net hydrogen, isoelectric point (pI), side bulk] and toxicity. For the hydropathicity, ToxinPred2.0 usually uses the classic Kyte–Doolittle index to calculate the hydrophilicity and hydrophobicity of each amino acid and then averages the entire peptide chain. The Kyte–Doolittle index for a single amino acid ranges from −4.5 (arginine) to +4.5 (isoleucine) [30]. We used the batch submission module prediction of peptides and the SVM-PROT (Swiss-Prot) method, where the E-value cut-off for the motif-based method is 10. ToxinPred2.0 was still used to predict the potential activity of the peptides, and peptides with potential activity scores greater than 0.80 were selected according to the ranking of the potential activity scores to undergo screening via the machine learning model for AutoDock Vina 1.5.7 [31] docking. A total of 101 peptides to be docked were screened.

### 2.5. Molecular Docking and Binding Analysis

Given the absence of a fully resolved DPP-4 structural model (particularly the transmembrane domain), we employed the AlphaFold sever (https://deepmind.google/technologies/alphafold/alphafold-server/, accessed on 17 January 2025) to predict the DPP-4 structure as our computational framework. We used PyMOL 3.0.4 manual linkage to generate 78 short peptides and PEP-FOLD4 (https://bioserv.rpbs.univ-paris-diderot.fr/services/PEP-FOLD4/, accessed on 17 January 2025) to generate peptides whose residues are more than 10, both saved as PDB files. Following that, we used AutoDock Tools 1.5.7 to perform the pre-docking processing, where the DPP-4 protein was dewatered, hydrogenated, small-molecule-removed and monomer-separated, the peptides were dewatered and hydrogenated, and the final output was a pdbqt file. We ran the AutoDock Vina interfacing program for the docking. The grid data (center x= −12.48, center y = 9.61, center z = 21.55, size x = 126.00, size y = 100.00, size z = 126.00) were selected and the docking was executed 10 times. PyMOL 3.0.4 and Discovery Studio 4.5 were used for visualization and the peptides as well as the DPP-4 monomers were separated into separate PDB files.

### 2.6. Molecular Dynamics Simulations

All the MD simulations and analyses were conducted using Amber 22 software [32]. The workflow began with the system preparation, where the ligand structure was protonated using the “reduce” tool and parameterized via the “antechamber” module with the AM1-BCC charge model, generating gaff2-compatibleforce field parameters validated by “parmchk2”. The receptor protein (DPP-4) was preprocessed with “pdb4amber” to standardize the hydrogen atom naming and remove any non-AMBER-compatible entries. The solvated complex was assembled in the “tleap” environment using the ff19SB force field [33] for the protein, TIP3P for the water model, and GAFF2 for the ligand. The system was neutralized with Na^+^/Cl^−^ ions and solvated in an octahedral water box with a 12 Å buffer, producing topology and coordinate files (“prmtop” and “inpcrd”) for the subsequent simulations.

The MD simulations were executed in four stages using the “pmemd.cuda” GPU-accelerated engine: energy minimization (5000 steps of the steepest descent and conjugate gradient), heating (0–300 K over 50 ps under the NVT ensemble with positional restraints), equilibration (100 ps NPT at 300 K and 1 bar), and production runs (100 ns unrestrained NPT with a 2 fs time step). The basis for choosing 100 ns for the simulation is that the RMSD of the short peptide binding/dissociation process in this experiment reaches equilibrium within 80–100 ns. The trajectories were processed using “CPPTRAJ”, where the solvent and ions were stripped for analysis, and structural metrics—including the root mean square deviation (RMSD) and root mean square fluctuation (RMSF) of the Cα atoms, radius of gyration (R_g_), solvent-accessible surface area (SASA) and secondary structure via the Definition of Secondary Structure of Proteins (DSSP) advanced analyses included principal component analysis (PCA) to identify the dominant conformational motions, k-means clustering (k = 10) based on the backbone RMSD to classify the structural states, and binding free energy calculations using the MM/PBSA.py script with the Poisson–Boltzmann (PB) methodology and residue-level decomposition (1000 frames sampled at 10-interval increments).

### 2.7. Materials

The four peptides (PPPPPPPPA, PPPSPPPV, PPPPPY, CPPPPAAY) with a purity of 95% were synthesized by Inner Mongolia Medical University (Figure S1). DPP-4 enzyme and Gly-Pro-pNA (substrate) are both derived from Adamas (Shanghai Adamas Reagent Co., Ltd., Shanghai, China).

### 2.8. Determination of DPP-IV-Inhibitory Activity

The assay method for measuring the peptide inhibition of DPP-4 was based on the combination of two established protocols [34,35]: the peptides were dissolved in ddH_2_O to prepare a gradient of concentrations and the DPP-4 enzyme was diluted in Tris–HCl (20 mM, pH 8.0). In a 96-well plate, 30 µL ddH_2_O, 10 µL peptide solution, 50 µL substrate (1 mM), and 10 µL DPP-4 enzyme (0.05 mg/mL) were added sequentially. Each peptide concentration was tested in triplicate and incubated at 37 °C in the dark for 15 min, and then 100 µL of 0.1 M NaHCO_3_ solution was added to stop the reaction. The absorbance at 405 nm was measured on a microplate reader, and the percent inhibition of DPP-4 was calculated at each concentration. The IC_50_ values for the four peptides were determined by fitting the dose–response curves with the log peptide concentration on the x-axis and the DI (%) on the y-axis. The DPP-IV-inhibition (DI) was calculated as follows:

(8) $$DI\left(\%\right)=(1-\frac{A_{405}\left(peptide\right)-A_{405}\left(buffer\right)}{A_{405}\left(positive\;control\right)-A_{405}\left(negtive\;control\right)})\times egt$$

where A_405_ (peptide) represents the optical density (at 405 nm) of the enzyme (DPP-IV) in the presence of the substrate (Gly-Pro-p-nitroanilide) and inhibitor (inhibitory peptide); A_405_ (buffer) represents the optical density of ddH_2_O; A_405_ (positive reaction) represents the optical density of the enzyme and the substrate in the absence of inhibitor; and A_405_ (negative reaction) represents the optical density of the enzyme in the absence of the substrate and inhibitor.

### 2.9. Statistical and Correlation Analysis

Origin 2024 was used to sort out the physical and chemical property data and create heatmaps to visualize the correlation between the peptide activity, the docking energy, and the physicochemical properties. GraphPad Prism 10 was used to perform non-linear regression of the DPP-4 percent inhibition versus the log[concentration]. The differences between the IC50 values of DPP-IV were subjected to the ordinary one-way ANOVA test. A *p*-value < 0.05 was considered significant [34]. Other images were created by Python 3.13.2 and Adobe Illustrator 2022. We evaluated Section 2 of the article using the TRIPOD (Transparent Reporting of a multivariable prediction model for Individual Prognosis Or Diagnosis) framework from the EQUATOR Network [36].

## 3. Results

### 3.1. Machine Learning Integration

**Figure 1:**
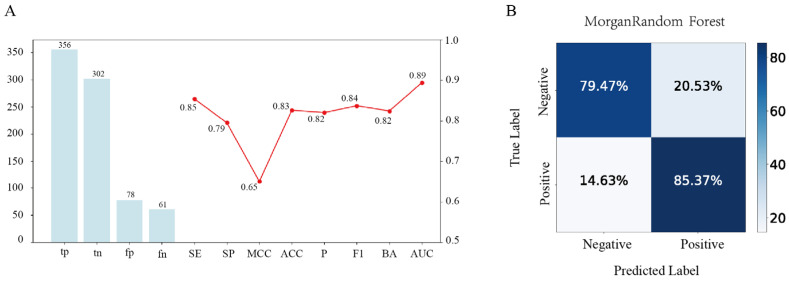
Performance evaluation of the Morgan-fingerprint-based random forest (Morgan–RF) model. (**A**) Combined metric visualization, where the bar chart (left y-axis) shows the confusion matrix counts for the Morgan–RF model, while the line chart (right y-axis) displays multiple performance metrics ranging from 0.50 to 1.00. (**B**) The dual-percentage confusion matrix presents the row- and column-wise percentages of the predicted versus true labels.

The Morgan–RF model demonstrates strong predictive performance, as evidenced by its multiple performance metrics and confusion matrix. The inclusion of multiple performance metrics in the line chart supports the robustness of the model, reinforcing its suitability for screening potential DPP-4 inhibitors. The model has outstanding performance in terms of the overall differentiation ability (AUC = 0.89), which is suitable for scenarios requiring high confidence ranking, such as drug candidate screening. However, there are some errors in the specific classification results (MCC = 0.65), which need to be further optimized to reduce the misjudgment (Figure 1A). The accompanying dual-normalized confusion matrix further emphasizes the model’s balanced prediction ability (Figure 1B).

### 3.2. Physicochemical Properties of Rice Peptides and Their Correlation

**Figure 2:**
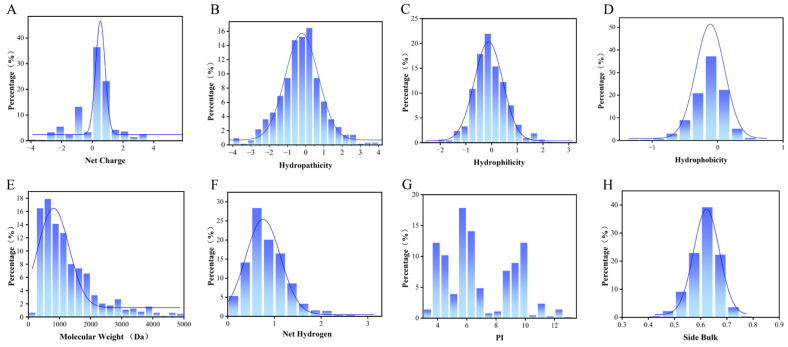
Distribution of the physicochemical properties of rice peptides predicted by computational methods. (**A**) Net charge, (**B**) hydropathicity, (**C**) hydrophilicity, (**D**) hydrophobicity, (**E**) molecular weight, (**F**) net hydrogen, (**G**) isoelectric point (pI), and (**H**) side bulk. “Percentage” denotes the proportion of rice peptides within this range. The blue curve represents the Kernel Density Estimation curve.

Rice peptides exhibit diverse charge states (positive, negative, or neutral) with a broad yet moderate charge distribution (Figure 2A). Rice polypeptides contain hydrophilic peptides and relatively more hydrophobic peptides (Figure 2C,D), which means that they may affect the DPP-4 inhibition in protein binding through different modes of action, such as hydrogen bonding or hydrophobic interaction. The molecular weight of the rice peptides was concentrated in the lower range, which was consistent with the characteristics of short bioactive peptides (Figure 2E). Low-molecular-weight peptides usually have good cellular permeability and easier access to the active cavity of DPP-4, which is conducive to the improvement of their bioavailability in vivo.

The distribution of the net hydrogen indicates that the polypeptide has a certain ability to form hydrogen bonds, and the number of hydrogen bonds affects the stability of the polypeptide and may also determine its affinity at the receptor binding site, which is crucial for DPP-4 binding (Figure 2F). Rice peptides have relatively low pI values (Figure 2G), where the pI determines the solubility and charge state of the polypeptide in different PH environments. In the range closest to physiological pH (7.4), the charged state of the peptide affects its electrostatic interaction with DPP-4. DPP-4 binding pockets are rich in Glu/Asp, so electrostatic action promotes the binding of the two. The distribution of the side bulk reflects the spatial adaptability of the polypeptides at the binding sites. The rice peptides concentrated between 0.60 and 0.65 (Figure 2H), and the volume was relatively large.

**Figure 3:**
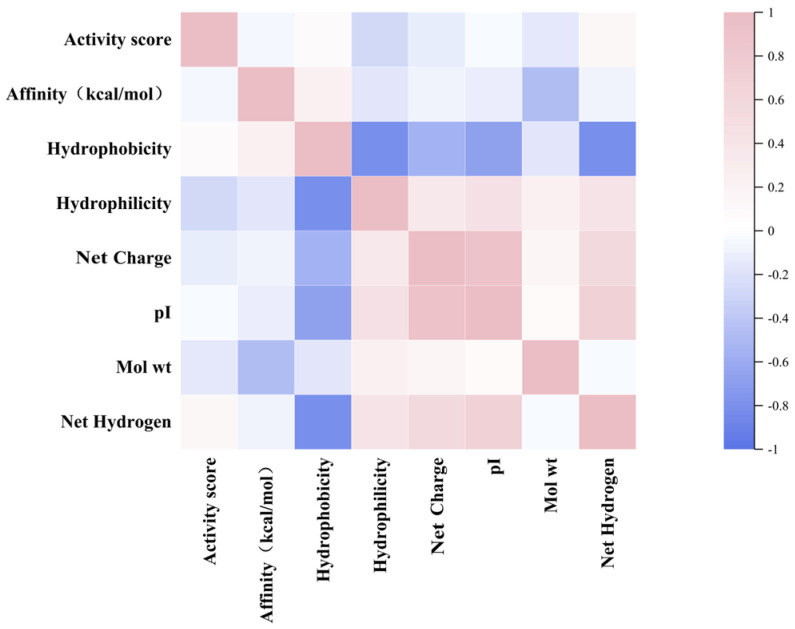
Heatmap showing the relationship between the activity score, the affinity and the physicochemical properties of rice peptides.

Based on the correlation analysis of the physicochemical properties and inhibitory potential, the binding affinities and activity scores of rice polypeptides were further explored, indicating that the hydrophobicity is weakly positively correlated with the binding affinity (0.22) and the net hydrogen is weakly positively correlated with the activity scores (0.13), suggesting that hydrophobic interaction plays a key role in DPP-4 binding and peptides with higher net hydrogen may have bioactivity to a certain extent. The net charge and pI are often correlated (0.91), reflecting the effect of the overall charge distribution on the binding ability of the peptide. The hydrophobicity has a strong negative correlation with the number of hydrogen bonds (−0.81), because hydrophobic polypeptides are usually rich in non-polar residues (such as alanine, etc.) and less polar residues (such as serine, glutamine, etc.). Since hydrogen bonds are mainly provided by polar residues, an increase in hydrophobic residues leads to a decrease in the number of groups that can participate in hydrogen bonds, resulting in a negative correlation. Hydrophobicity drives the polypeptide to fold and reduce its exposure to polar groups, while hydrogen bond formation depends on the availability of polar groups and solvent accessibility (Figure 3).

### 3.3. Docking Visualization and Peptide Analysis

The AlphaFold-predicted structures offer a detailed view of DPP-4’s interactions with four selected rice peptides (PPPPPPPPA, PPPSPPPV, PPPPPY, CPPPPAAY) and Diprotin A (IPI). According to the 2D diagram, the four peptides form fewer bonds with DPP-4 compared to IPI, and the interacting residues involved differ significantly. The interactions between IPI and DPP-4 are predominantly carbon–hydrogen bonds, whereas the four peptides bind to DPP-4 through mixed interactions. Hydrogen bonds, salt bridges, and various hydrophobic interactions elucidate the molecular basis of the binding stability. The presence of conventional and carbon–hydrogen bonds suggest strong interactions, while the alkyl and π-sigma interactions further contribute to the peptide docking efficacy. Some “unfavorable bonds” (marked in red) reflect the presence of spatial exclusion or a mismatch at the polypeptide binding interface with DPP-4.

**Figure 4:**
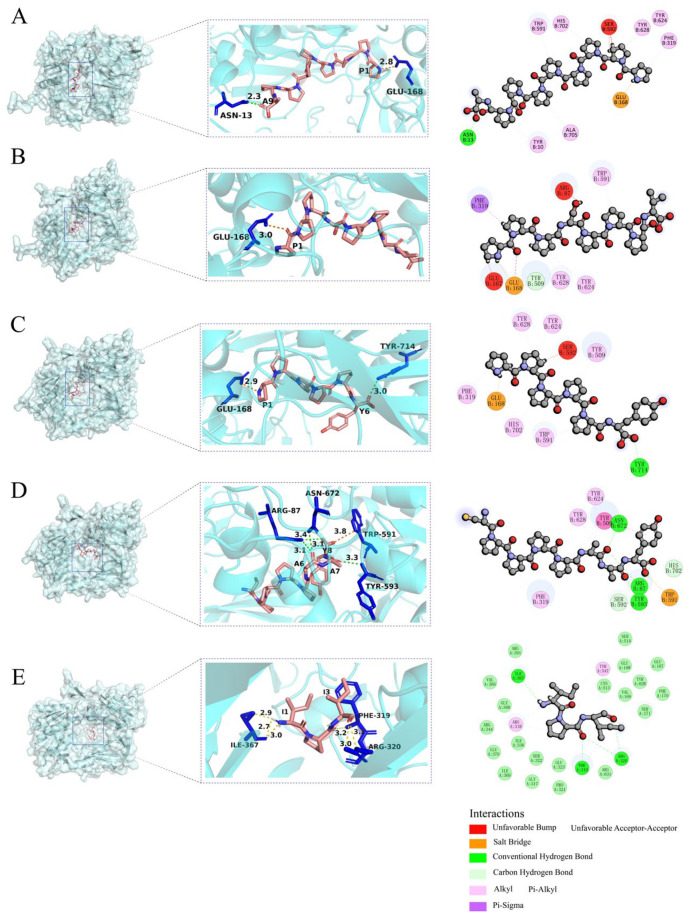
AlphaFold-predicted binding models of DPP-4 (semitransparent blue surface) with (**A**) PPPPPPPPA, (**B**) PPPSPPPV, (**C**) PPPPPY, (**D**) CPPPPAAY, and (**E**) IPI. Hydrogen bonds are denoted by yellow dashed lines, salt bridge are shown by orange dashed lines, peptide residues are labeled using single-letter abbreviations, while DPP-4 residues are indicated with three-letter abbreviations in the 3D interaction diagrams.

The 3D molecular structure analysis showed that the polar or charged residues in DPP-4, such as Arg and Glu, are more likely to participate in hydrogen bonding and salt bridge formation with polypeptides. The flexibility and exposure of the C-terminal and N-terminal residues of the peptide make it easier to access and form hydrogen bonds and salt bridges with the key binding sites of DPP-4. In addition, all four peptides are rich in proline, which is also consistent with the characteristic that DPP-4, as a peptidase, is sensitive to the N-terminal sequence of polypeptides, especially Pro. This adverse interaction suggests that the conformation or side chain arrangement of the peptides in this region is not optimally complementary to the DPP-4 surface, which is a potential target for subsequent sequence optimization (Figure 4).

The hydrophobicity and interpolated charge distribution across the peptide regions critically influences the docking efficacy. Strongly hydrophobic regions facilitate stable hydrophobic interactions with DPP-4′s nonpolar residues (e.g., Phe319). High-charge-density regions help to bind to oppositely charged residues in DPP-4 by electrostatic action, such as salt bridges, thereby enhancing the affinity.

**Figure 5:**
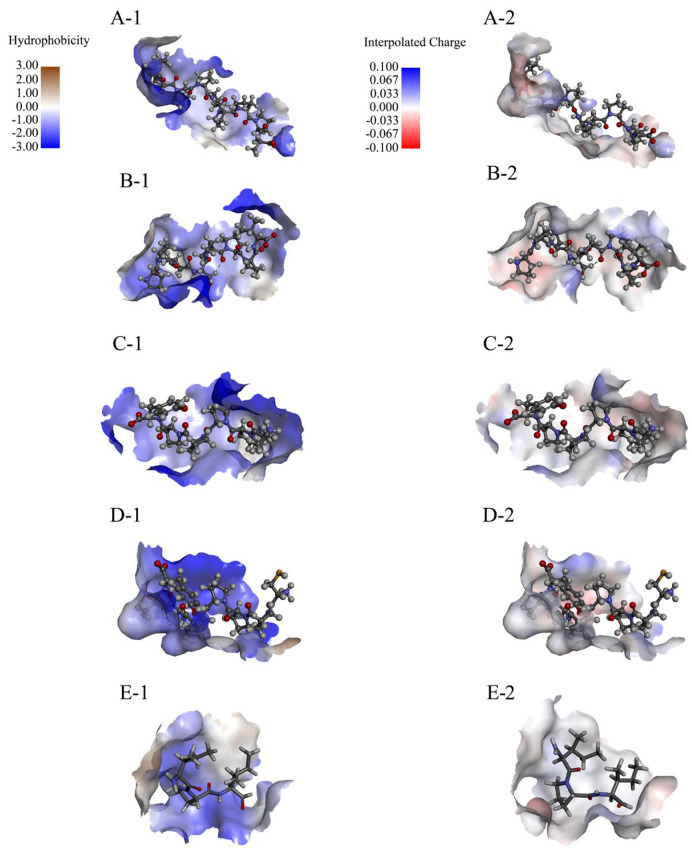
Hydrophobicity and interpolated charge of the AlphaFold-predicted rice peptides. (**A-1**,**B-1**,**C-1**,**D-1**,**E-1**) represent the hydrophobicity range of PPPPPPPPA, PPPSPPPV, PPPPPY, CPPPPAAY, and IPI; and (**A-2**,**B-2**,**C-2**,**D-2**,**E-2**) represent the interpolated charge range of PPPPPPPPA, PPPSPPPV, PPPPPY, CPPPPAAY, and IPI.

Compared with IPI, the binding peptides have a wide distribution of interpolated charge and an alternating distribution of positive and negative charge residues (Figure 5). The alternating distribution of positive and negative charge residues can avoid aggregation or precipitation caused by single charge enrichment. It also reveals that the peptide may stabilize the β-sheet structure or inhibit the α-helix (electrostatic repulsion within the helix) by electrostatic complementation between adjacent chains when interacting with DPP-4, but the specific conformation selection depends on the subsequent DSSP analysis.

### 3.4. Integrated Characterization of Structural Stability, Flexibility, and Ligand Binding Properties in Four Molecular Systems

#### 3.4.1. Structural Stability and Global Conformational Features Across Five Molecular Systems

Based on the multi-parametric molecular dynamics analyses, the conformational stability and structural compactness of the five peptide-DPP-4 complexes are characterized as follows.

**Figure 6:**
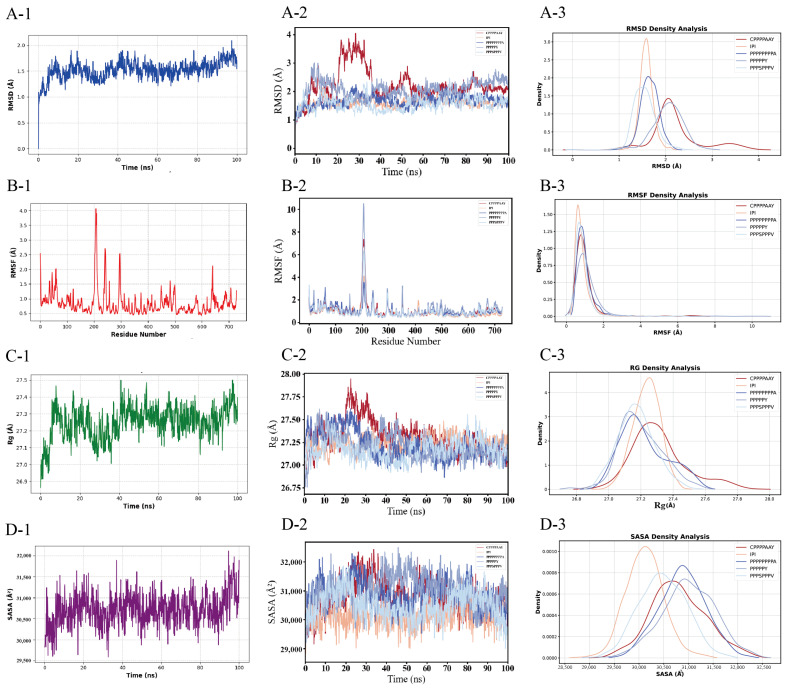
Temporal evolution and statistical distribution analyses of the molecular dynamics parameters for simulated systems. (**A**) Root mean square deviation (RMSD), (**B**) root mean square fluctuation (RMSF), (**C**) radius of gyration (R_g_), and (**D**) solvent-accessible surface area (SASA). Left panels (**A-2**,**B-2**,**C-2**,**D-2**) display the time-dependent trajectories of each parameter, whereas right panels (**A-3**,**B-3**,**C-3**,**D-3**) show their normalized probability density distributions. Distinct colors correspond to the four simulation systems. Panels (**A-1**,**B-1**,**C-1**,**D-1**) quantify the baseline RMSD, RMSF, R_g_, and SASA values for monomeric DPP-4 in its unbound state.

First, the RMSD of all five systems evolves toward stability over time (final values < 2.80 Å), indicating that after binding, the complexes have reached an energy-converged state with no significant conformational drift (Figure 6(A-2)). Specifically, during the initial binding phase (0–40 ns), the RMSD increases rapidly (Δ ≈ 3.00 Å). In the equilibrium phase (>60 ns), the RMSD fluctuations decrease to approximately 0.50 Å, suggesting that a stable binding mode has been established. Secondly, the radius of gyration (R_g_) of the five systems decreases upon binding, with the difference being less than 0.30 Å (from 27.50 Å to 27.20 Å) (Figure 6(C-2)), indicating that binding does not induce a substantial overall deformation of the protein. However, peptide binding alters the upward trend in the R_g_ observed in native DPP-4 (Figure 6(C-1)), implying that peptide binding induces a more compact overall structure in DPP-4.

#### 3.4.2. Residue-Specific Flexibility and Solvent Accessibility Dynamics

According to the UniProt P27487 sequence DBREF mapping, the catalytic triad of DPP-4 is unequivocally identified as Ser592–Asp670–His702 in this study. The RMSF of the residues within the S1/S2 subpockets and surrounding regions (Trp591–His702) of DPP-4 exhibits a decreasing trend upon binding (<1.75 Å compared to nearly 2.20 Å in the unbound state) (Figure 6(B-1,B-2)). Specifically, the hydrophobic residues (Pro) in the peptide form stacking interactions with Phe319, Tyr624, and Tyr628 in the S2 pocket to restrict local motion, while the Pro residue also forms salt bridges with Glu in DPP-4, thereby suppressing the conformational fluctuations of the catalytic triad Ser592–Asp670–His702 (Figure 4 and Figure 6(B-2)) and reducing the local flexibility. Following binding, the SASA at the peptide–protein interface gradually decreases (from 32,500 Å^2^ to 31,500 Å^2^). Among them, the SASA of the IPI is lower than that of the four potential inhibitory peptide systems, indicating an increased burial of the hydrophobic core and/or polar residues (Figure 6(D-2)). At this stage, the peptide progressively buries key residues into the DPP-4 binding pocket through conformational adjustments. Compared with unbound DPP-4 (SASA ≈ 32,800 Å^2^), the SASA of the complex system is reduced by approximately 1300 Å^2^ (Figure 6(D-1)), thereby confirming a significant interfacial burial effect induced by peptide binding.

Binding density analysis of the five systems reveals a structure–activity relationship model. PPPSPPPV exhibits exceptionally high structural stability (rigid architecture), low global fluctuations, absence of metastable states, and low SASA, although its stability and burial remain lower than those of IPI. PPPPPY displays moderate stability with high conformational heterogeneity, flexible functional regions, and fluctuating SASA. PPPPPPPPA shows moderate-to-low stability with localized unfolding propensity and intermediate SASA. CPPPPAAY demonstrates moderate stability with ligand-induced conformational contraction and specific solvent exclusion (Figure 6(A-3)–(D-3)).

#### 3.4.3. Conformational Landscape Evolution and Secondary Structure Stability

Based on PCA, k-means clustering, and DSSP, coupled with the catalytic triad, the dynamic evolution of the secondary structure, and motion correlation analysis, it is evident that DPP-4 precisely regulates the conformation of its catalytic domain through dynamic reconfiguration of its α-helix, 3_10_-helix, and π-helix. Furthermore, the binding efficacy of the peptide inhibitors depends on the stability of the induced secondary structure and the corresponding motion correlation patterns.

**Figure 7:**
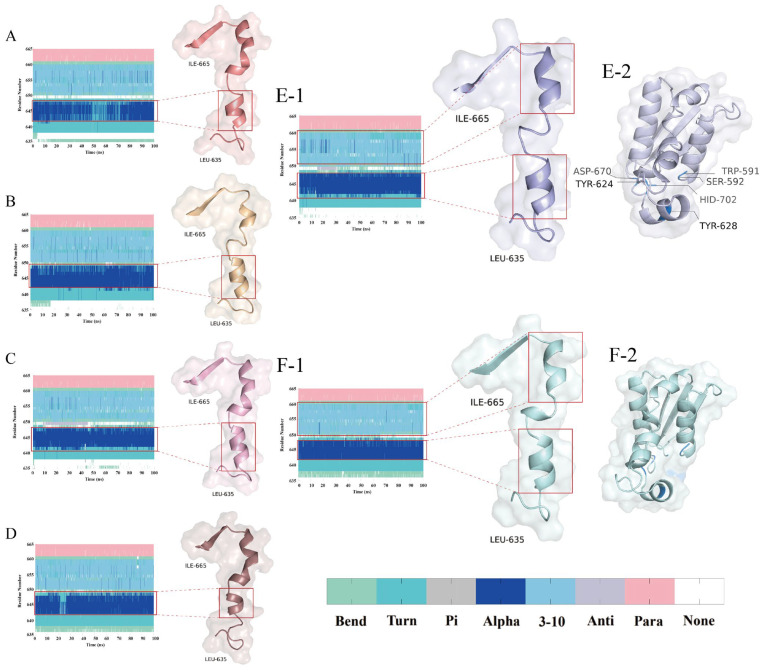
Temporal evolution of the secondary structural conformations and DPP-4 structural analysis. (**A**–**D**,**E-1**,**F-1**) display the DSSP-derived secondary structure dynamics for the PPPPPPPA, PPPSPPPV, PPPPPY, CPPPPAAY, apo-DPP-4 (unbound state) and IPI systems across regions Leu635–Ile665; and (**E-2**,**F-2**) display the non-bond and IPI structure Trp591–His702. Secondary structure codes: None (disordered), Para (parallel β-sheet), Anti (antiparallel β-sheet), 3-10 (3_10_-helix), Alpha (α-helix), Pi (π-helix), Turn (β-turn), and Bend (random coil).

The α-helix (Asn641–Asn647) exhibits an elongation trend across the four systems, with the number of residues increasing sequentially from system A to D (four, four, five, and four, respectively, compared to an initial count of five in native DPP-4) (Figure 7(A–D,E-1)). This change shortens the distance between the catalytic domain and the substrate and optimizes the attack angle, thereby significantly enhancing the rigidity of the binding interface. The 3_10_-helix (Thr649–Gln659) is stably present in systems B and C (comprising 11 residues), and its dynamic extension and contraction—through modulation of the side-chain orientation of Tyr624 (with an RMSF reduction of 20.00%)—maintains the stability of the hydrophobic core. In addition, in system C, Thr649 briefly adopts a π-helix conformation during the 10–40 ns window (Figure 7(C-1)), suggesting that DPP-4 undergoes local conformational rearrangements in the early binding phase to select the optimal binding state.

**Figure 8:**
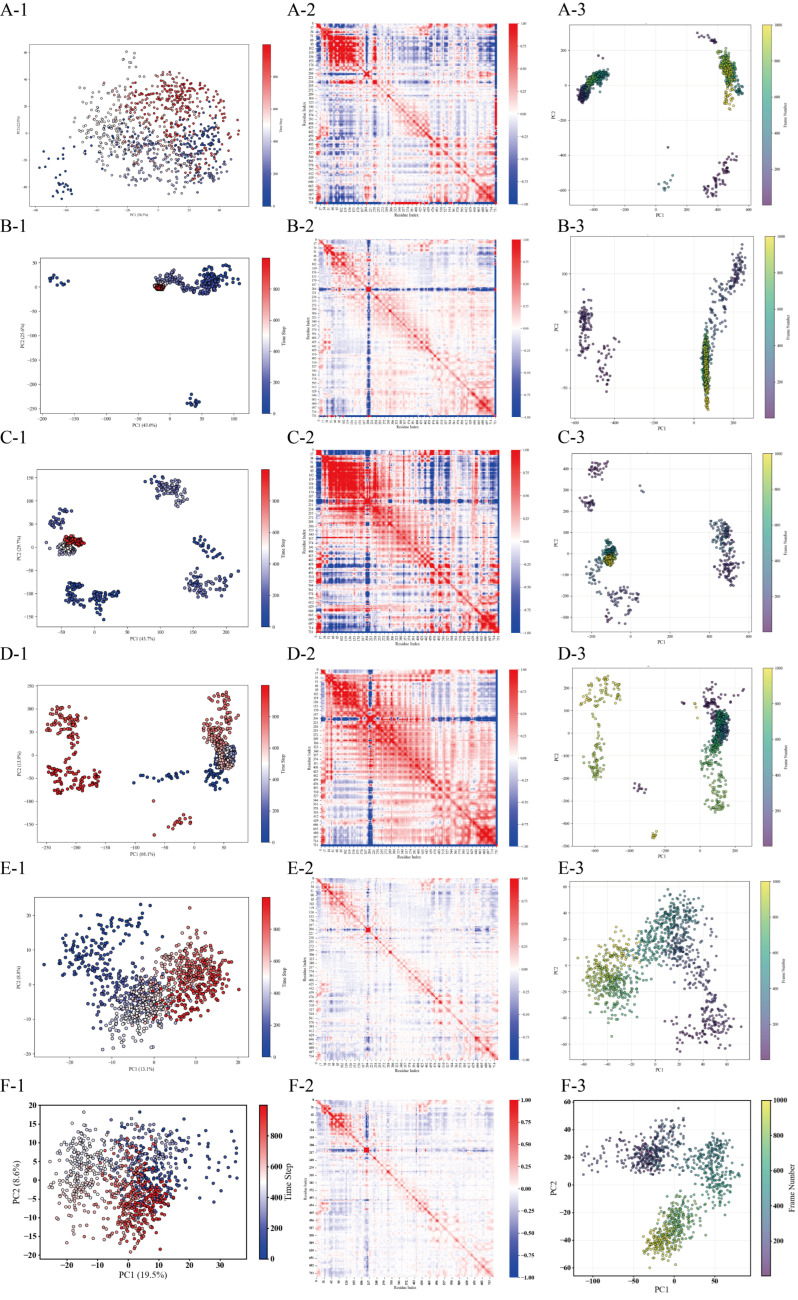
Conformational landscape analysis of four DPP4-peptide complexes via PCA and k-means clustering. Systems: PPPPPPPA (**A**), PPPSPPPV (**B**), PPPPPY (**C**), and CPPPPAAY (**D**), apo-DPP-4 (unbound state, **E**) and IPI (**F**). Each system contains three panels: (X-1) k-means clustering results (colored/shaped points = clusters) projected onto the first two principal components; (X-2) heatmap of the feature index hierarchy (red-blue gradient) aligned with the (X-1) cluster groups; and (X-3) alternative PCA visualization with the frame number represented by color gradients.

Considering its low RMSD fluctuations, low RMSF, and low variance contributions of PC1/PC2 (52.50%) (Figure 6 (A-2,B-2) and Figure 8(A-1)), it appears that the principal components in system A have not captured the primary modes of motion adequately, and further analysis of the higher-order principal components (PC3 and beyond) is warranted to capture the dynamic coupling among the distal domains. In contrast, system B exhibits three distinct conformational clusters that reflect the dynamic process of peptide binding (binding–adjustment–stabilization). The time steps, ranging from 500 ns to 1000 ns, show data points that remain contiguous without abrupt jumps, indicating relative conformational stability (Figure 8(B-1)). System D, however, exhibits considerable fluctuations in both the conformation and the RMSD; around 200 ns, the data points display significant displacements, and the RMSF also shows large fluctuations, suggesting that the weak interactions between the peptide and DPP-4 lead to frequent conformational adjustments (Figure 6 (B-2) and Figure 8(D-1)).

The covariance matrix displays the degree of motion correlation among the Cα atoms. In system B, the matrix shows high correlation around the diagonal, with weaker correlation in regions farther from the diagonal (Figure 8(B-2)), indicating that the primary motions are localized and that the overall system is ordered and stable. Overall, the high similarity in terms of the correlation between system B and DPP-4 supports the functional advantage of system B (Figure 8(E-2)). Systems A and C, on the other hand, exhibit pronounced mixed positive and negative correlations in certain regions, suggesting the presence of larger conformational changes or more complex cooperative motion patterns (Figure 8(A-2,C-2)).

Analysis of the principal component projections (Mode1 and Mode2 from the PCA results) allows observation of the evolution of the protein–peptide systems under the two most fundamental primary vibrational modes. Comparing A-3, B-3, C-3, and D-3 (Figure 8) reveals that system B displays a relatively compact distribution; in conjunction with the DSSP analysis, it is evident that the secondary structures (α-helix and 3_10_-helix) near the catalytic triad in system B remain intact, further confirming its stability. Systems A and C exhibit more pronounced multi-cluster or transitional trajectories, indicating significant conformational changes during the simulation, while system D’s distribution lies between these two cases.

#### 3.4.4. Thermodynamic Profiling of Ligand Binding Interactions

The molecular mechanics/Poisson–Boltzmann surface area (MM/PBSA) energy decomposition reveals differences in the binding free energy as well as the contributions from key residues. Based on the MM-PBSA binding free energy calculations and residue energy decomposition, the thermodynamic characteristics of the binding and the key interaction sites between the four peptide systems and DPP-4 can be systematically summarized as follows.

The total binding free energy of systems A, B, and C are all lower than that of the positive control group (IPI). System B exhibits highly efficient binding driven by a synergistic interplay between the polar and hydrophobic interactions, with a thermodynamic advantage (ΔTOTAL) of –29.22 kcal/mol—significantly lower than the other systems, indicating the highest binding affinity. In this system, polar interactions dominate (EEL = −123.82 kcal/mol, accounting for 80%), where the peptide’s Pro residue forms a stable salt bridge with the Glu168 in the S2 hydrophobic pocket of DPP-4; hydrophobic interactions further contribute (VDWAALS = −31.63kcal/mol) via the close packing between the Pro side chain of the peptide and Phe319 in the S2 pocket. Additionally, Tyr624 and Tyr628 interact with His702 in the S1 pocket through Pi–alkyl interactions and hydrogen bonds, thereby locking the orientation of the catalytic triad (Figure 4C). Moreover, the low standard error (±0.92 kcal/mol) and RMSF peak values (<7.00 Å) confirm that the binding interface undergoes minimal dynamic fluctuations, with a rigid hydrophobic core and a relatively stable conformation. In contrast, system A’s binding advantage is weakened by solvation losses. Although the gas-phase free energy (∆G_gas_ = −191.97 kcal/mol) indicates strong attractive forces, the overall ΔTOTAL is only –20.64 kcal/mol, signifying that the desolvation penalty (∆G_solv_ = +171.33 kcal/mol) substantially offsets the binding advantage. This energy imbalance may be attributed to the excessive exposure of polar groups, as the peptide’s C-terminal carboxyl group is not effectively buried during solvation—leading to a loss of polar solvation energy. It may also result from suboptimal hydrophobic matching, as evidenced by a VDWAALS contribution (−14.98 kcal/mol) that is approximately 12% lower than that in system B, reflecting a geometric mismatch between the alkyl side chain and the S2 pocket. Notably, however, the RMSF in this system demonstrates an absolute advantage (with peak values below 4.00 Å). System D exhibits low binding efficiency (∆TOTAL = −10.12 kcal/mol) along with significant conformational fluctuations. Its insufficient hydrophobic interactions, as indicated by a markedly lower VDWAALS (–9.99 kcal/mol) compared to system B, suggest that the repetitive Pro residues fail to effectively occupy the S2 pocket; the RMSF peak values (>7.00 Å) further indicate frequent dynamic rearrangements at the binding interface (Table 1).

### 3.5. DPP-4 Inhibitory Profiles of Four Synthetic Peptides

**Figure 9:**
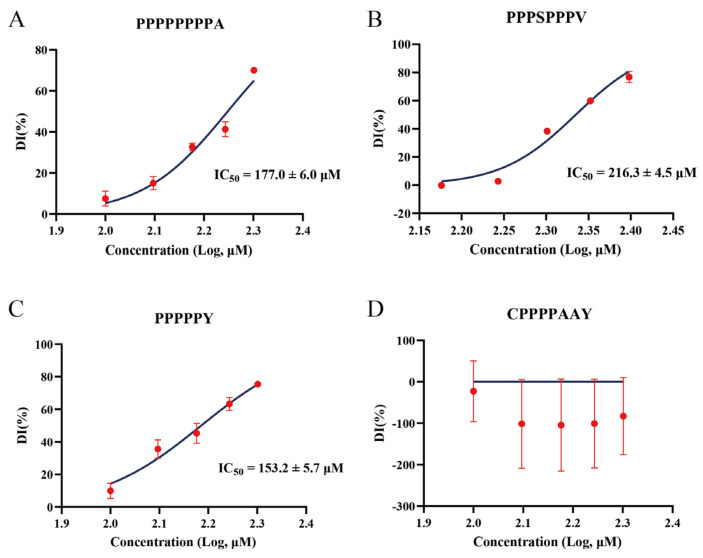
Concentration-dependent inhibition of DPP-4 by four synthetic peptides. (**A**–**D**) Dose–response curves for PPPPPPPPA (**A**), PPPSPPPV (**B**), PPPPPY (**C**), and CPPPPAAY (**D**) showing the percent of DPP-4 inhibition (DI%, mean ± SD, n = 3) at varying peptide concentrations (x-axis as log_10_ [μM]). Non-linear regression (curve fit) of a four-parameter logistic model (solid blue lines) yielded the IC_50_ values. CPPPPAAY exhibited no significant inhibition within the tested concentration range, and its IC_50_ could not be determined.

A preliminary screening to determine the effective concentration range of the four synthetic peptides against DPP-4 revealed that CPPPPAAY did not exhibit significant inhibition within the tested range. Subsequently, dose–response analyses were performed on all four peptides, demonstrating that three of them inhibited the DPP-4 activity in a concentration-dependent manner, whereas CPPPPAAY showed no inhibitory effect (Figure 9D). PPPSPPPV produced characteristic sigmoidal inhibition curves, 216.3 ± 4.5 μM, PPPPPPPPA has a superior IC_50_ compared to PPPSPPPV, at 177.0 ± 6.0 μM, respectively (Figure 9A,B), indicating moderate potency. In contrast, the shorter peptide PPPPPY was the most effective inhibitor, with an IC_50_ of 153.2 ± 5.7 μM (Figure 9C).

## 4. Discussion

### 4.1. Structural and Physicochemical Determinants of DPP-4 Inhibition

Inhibiting DPP-4 remains an established strategy for glycemic control in type 2 diabetes [37]. Rice-derived peptides like PPPSPPPV offer superior safety and mechanistic precision by synergizing electrostatic anchoring (Glu168 salt bridge) with hydrophobic S2 pocket occupation (Phe319/Tyr628 π-stacking) [38].

The physicochemical properties of rice peptides critically correlate with their inhibitory potency. Rice peptides show biological activity with wide net charge, higher hydrophobicity, good hydropathicity, low molecular weight and pI, providing rich candidates for screening effective DPP-4 inhibitors. Among them, the hydrophilic/hydrophobic balance and hydrogen bond formation ability can be the key factors affecting DPP-4 binding. The low molecular weight and wide charge distribution suggest that some peptides may have good bioavailability and be suitable candidates for natural DPP-4 inhibitors. By further optimizing these properties (such as adjusting the hydrophobicity, charge, or side chain volume), the effectiveness of rice peptides in terms of DPP-4 inhibition can be improved. This analysis provides a clear direction for subsequent activity verification and structure optimization, and it can also be used to design more targeted DPP-4 inhibitory peptides.

The correlation between the physicochemical properties of rice peptides and the binding affinity and activity scores was not obvious, but the above conclusions still help to clarify which parameters should be the focus of optimization and which can be appropriately ignored in subsequent screening.

### 4.2. Conformational Dynamics and Peptide Binding

The DPP-4 binding pocket is rich in acidic residues (e.g., Glu/Asp), enabling electrostatic enhancement for positively charged peptides (e.g., PPPSPPPV forming a salt bridge with Glu168), while neutral peptides rely on hydrophobic interactions (e.g., Pro stacking with Phe319/Tyr628). The diversity in the charge distribution (Figure 2A) suggests potential multi-target binding modes. The dual hydrophilic–hydrophobic modality (Figure 2C,D) and docking visualizations (Figure 4) support the “dual-pocket occupancy” theory [39], wherein hydrophilic groups (e.g., Tyr) anchor the catalytic domain via hydrogen bonds, while hydrophobic groups (e.g., Pro) occupy the S2 subpocket. Notably, the strong negative correlation between the hydrophobicity and the hydrogen bond count (r = −0.81) reflects a “polarity-hydrophobicity trade-off” in peptide design: excessive hydrophobicity (e.g., system D, CPPPPAAY) enhances hydrophobic matching but sacrifices polar complementarity, leading to solvation penalty (∆G_solv_ = +171.33kcal/mol). Thus, optimizing the hydrophilic–hydrophobic residue ratio (e.g., Pro/Ser alternation in system B, PPPSPPPV) is key to improving the inhibitory efficacy.

Helical dynamics are central to catalytic domain conformational regulation. PCA and secondary structure evolution reveal that DPP-4 modulates the spatial orientation of its catalytic domain through the dynamic extension/contraction of α-helices, 3_10_-helices, and π-helices (e.g., Asn641–Asn647 helix elongation shortening substrate attack distance). System B (PPPSPPPV) exhibits superior binding interface rigidity due to the stable retention of the catalytic domain-proximal α-helices (7 residues) and 3_10_-helices (11 residues), supported by its covariance matrix showing localized ordered motions (Figure 8(B-1)). These findings validate the hypothesis that “specific amino acids within helical conformations determine the aminopeptidase function of DPP-4” [40] and propose a novel design strategy: enhancing catalytic domain locking via optimized helical interaction networks.

This study systematically deciphers the molecular mechanism of peptide-mediated DPP-4 inhibition from the perspectives of dynamic conformational evolution and energy synergy, establishing that the high efficacy of the PPPSPPPV system arises from its tripartite mechanism (“rigid interface–helix stabilization–polar-hydrophobic synergy”).

Collectively, this work provides a universal strategy for designing peptide drugs targeting the DPP-2/8/9 family and underscores the pivotal role of dynamic structure–activity analysis in rational drug development.

### 4.3. Analysis of Rice-Derived Peptide Inhibitors Relative to IPI

When using the known DPP-4 inhibitory peptide IPI (IC_50_ ≈ 3.5 μM) as a positive control, the three peptides identified in this study exhibited 40–60 times lower inhibitory activity compared to IPI, consistent with the established mechanism by which IPI fits efficiently into the DPP-4 active site [41,42]. The IC_50_ values of the three peptides were 153.2 ± 5.7 μM for PPPPPY, 177.0 ± 6.0 μM for PPPPPPPPA, and 216.3 ± 4.5 μM for PPPSPPPV (Figure 9A–C). The inhibitory potency increased as the peptide length decreased and the proline density increased, suggesting that PPPPPY has the most favorable fit within the DPP-4 active site. In contrast, the additional residues in the longer peptides may introduce steric hindrance, weakening the binding affinity. Overall, these findings demonstrate that virtual hydrolysis of rice proteins can yield bioactive peptides with DPP-4 inhibitory potential, offering a promising strategy for the development of novel food-derived DPP-4 inhibitors.

### 4.4. Limitations and Future Directions

Whether the rice peptides obtained through virtual enzymatic hydrolysis using ExPASy-Peptide Cutter can truly be produced by hydrolyzing rice protein powder solutions with the corresponding enzymes remains uncertain. This can only be verified by dissolving rice protein powder, hydrolyzing it with the relevant enzymes, and analyzing the products via high-performance liquid chromatography (HPLC) or mass spectrometry. However, we selected Unipept (https://unipept.ugent.be/mpa, accessed on 18 May 2025) to identify the lowest common ancestor (LCA) of the four screened peptides. The results showed that the LCA of these peptides was *Oryza sativa* (rice), which provides partial validation of their origin. Although the RMSD analysis of the six systems indicated equilibrium after 100 ns simulations, these simulations might still fail to fully capture the long-term conformational changes in DPP-4 upon peptide binding. Extending the simulation durations or employing enhanced sampling techniques could address this. Additionally, this study lacks clinical trials, making it difficult to assert the clinical therapeutic potential of the screened peptides. The feasibility of these peptides for treating type 2 diabetes requires further clinical validation.

### 4.5. Possible Clinical Perspectives

DPP-4 inhibitory peptides, such as the PPPPPY screened in this study, may exhibit weight-neutral properties, making them suitable for obese patients with type 2 diabetes. Obesity is a major comorbidity of type 2 diabetes, and certain glucose-lowering drugs (e.g., insulin, sulfonylureas) may induce weight gain [43]. Natural peptides such as DPP-4 inhibitors could avoid weight gain and indirectly improve metabolism by modulating the GLP-1 pathway. Plant-derived peptides may achieve mild glucose-lowering effects through multi-target effects (e.g., stabilizing α/3_10_ helices, inhibiting catalytic triads), reducing the hypoglycemia risks. Although traditional DPP-4 inhibitors (e.g., sitagliptin) are relatively safe, rare adverse effects like pancreatitis and arthralgia persist [5]. Natural peptides, with their simple structures and lack of chemical modifications, may lower such risks.

DPP-4 may participate in COVID-19 infection, and its inhibitors might mitigate inflammatory responses via immunomodulation [44]. Diabetic patients face higher risks of severe COVID-19 outcomes, and DPP-4 inhibitors’ anti-inflammatory effects could improve their prognosis.

Synthetic drugs often face adherence limitations due to side effects, whereas plant-based peptides could be developed as functional foods or adjuvant therapies. Rice protein hydrolysates, as natural products, may reduce immunogenicity and suit long-term use [15]. Rice, a staple in Asian diets, allows integration of its protein peptides into daily nutrition as a culturally adapted diabetes management strategy. Dietary intervention is an adjunct therapy for type 2 diabetes, where matching insulin doses to carbohydrate, fat, and protein intake during meals remains critical [5]. Functional foods refer to those that provide potential health benefits beyond their nutritional value. Epidemiological studies highlight the importance of dietary foods in managing and reducing diabetes-related complications. Regular consumption of these dietary foods may aid blood glucose control, pancreatic β-cell function, insulin secretion, and weight management [15]. The use of rice peptides as dietary supplements could seamlessly align with Asian dietary habits, enhancing patient compliance. Peptide-based drugs with higher potency and specificity are expected to emerge in the near future.

## 5. Conclusions

This study successfully identified PPPPPY and PPPSPPPV as two promising DPP-4 inhibitory peptides through an AI-assisted virtual screening pipeline integrating machine learning, molecular docking, and molecular dynamics simulations. Among them, PPPPPY exhibited the strongest in vitro inhibitory activity, with an IC_50_ of 153.2 ± 5.7 μM. PPPSPPPV demonstrated strong binding free energy (ΔG ≈ –29.22 kcal/mol) and effectively stabilized the DPP-4 catalytic triad (Ser592–Asp670–His702) with low structural fluctuations (RMSF < 1.75 Å). Its binding mechanism involved electrostatic interaction with Glu168 and hydrophobic contacts with Phe319 and Tyr628. This study established an integrated structure–dynamics–activity framework for the discovery of natural DPP-4 inhibitors. Compared with synthetic drugs, rice-derived peptides may offer better biocompatibility and reduced side effects. Future research should focus on enzymatic validation, metabolic stability evaluation, and preclinical studies to support their application in health products or pharmaceutical development. 

## Figures and Tables

**Table 1:** Comparison table of the MM/PBSA data for the five systems from amber.

PEPTIDE	PPPPPPPPA	PPPSPPPV	PPPPPY	CPPPPAAY	IPI
TOTAL	−856.83 ± 1.60	−804.88 ± 2.94	−626.21 ± 1.36	−904.46 ± 1.02	−130.51 ± 0.81
VDWAALS	−14.98 ± 1.64	−31.63 ± 1.24	−23.39 ± 1.51	−9.99 ± 1.85	−35.79 ± 0.79
EEL	−176.99 ± 4.99	−123.82 ± 4.03	−111.43 ± 8.87	−89.09 ± 14.64	−54.15 ± 3.41
EPB	171.33 ± 4.58	126.24 ± 4.10	112.07 ± 6.48	88.96 ± 14.32	71.30 ± 3.54
DELTA G gas	−191.97 ± 6.11	−155.45 ± 4.12	−134.83 ± 9.34	−99.08 ± 15.96	−89.94 ± 3.71
DELTA G solv	171.33 ± 4.58	126.24 ± 4.10	112.07 ± 6.48	88.96 ± 14.32	71.30 ± 3.54
DELTA TOTAL	−20.64 ± 1.65	−29.22 ± 0.92	−22.75 ± 3.23	−10.12 ± 1.75	−18.64 ± 0.80

Note: The table summarizes the MM/PBSA-derived energy components of five peptide systems: A (PPPPPPPA), B (PPPSPPPV), C (PPPPPY), D (CPPPPAAY), and E (IPI), including the total system energy and decomposed terms: Van der Waals interactions (VDWAALS), electrostatic energy (EEL), polar solvation energy (EPB), gas-phase binding free energy (DELTA G_gas_, ΔG_gas_), solvation free energy (DELTA G_solv_, ΔG_solv_), and total binding free energy (DELTA TOTAL, ΔTOTAL). All the values are reported in kcal/mol.

## Data Availability

The data presented in this study are available in [https://github.com/582603/article, accessed on 28 May 2025] at [GitHub]. All the codes are available upon reasonable request from the authors. The software packages used in this study include RDKit, AutoDock Vina 1.5.7, PyMOL 3.0.4, and Amber 22. These data were derived from the following resources available in the public domain: PubMed (https://pubmed.ncbi.nlm.nih.gov/), PubChem (https://pubchem.ncbi.nlm.nih.gov/), ChEMBL (https://www.ebi.ac.uk/chembl/), BindingDB (https://www.bindingdb.org/), NCBI (https://www.ncbi.nlm.nih.gov/), ExPASy-Peptide Cutter (https://web.expasy.org/peptide_cutter/), ToxinPred2.0 (https://webs.iiitd.edu.in/raghava/toxinpred/), AlphaFold sever (https://deepmind.google/technologies/alphafold/alphafold-server/), PEP-FOLD4 (https://bioserv.rpbs.univ-paris-diderot.fr/services/PEP-FOLD4/).

## Supplementary Materials

The following supporting information can be downloaded at: https://www.mdpi.com/article/10.3390/foods14111916/s1, Figure S1. MALDI-TOF-MS of four synthetic peptides. PPPPPPPPA (A), PPPSPPPV (B), PPPPPY (C), and CPPPPAAY (D); Table S1. Oryza sativa glutelin and prolamin data from NCBI; Table S2. Expasy—Peptide Cutter Simulated Hydrolysis Results.

## Abbreviations

ACC	Accuracy
AUC	Area under the ROC curve
BA	Balanced accuracy
DELTA Ggas	Gas-phase binding free energy
DELTA Gsolv	Solvation free energy change
DELTA TOTAL	Total binding free energy
DPP-4	Dipeptidyl peptidase-4
DSSP	Definition of Secondary Structure of Proteins
EEL	Electrostatic energy (Coulombic interactions)
EPB	Electrostatic contribution to solvation (Poisson–Boltzmann)
F1	F1 score
GIP	Glucose-dependent insulin polypeptide
GLP-1	Glucagon-like peptide-1
HPLC	High-performance liquid chromatography
LCA	Lowest common ancestor
MCC	Matthews correlation coefficient
MD	Molecular dynamics
ML	Machine learning
MM/PBSA	Molecular mechanics/Poisson–Boltzmann surface area
P	Precision
PB	Poisson–Boltzmann
PCA	Principal component analysis
PDB	Protein Data Bank
Pdbqt	Protein Data Bank, Partial Charge (Q), and Atom Type (T)
pI	Isoelectric point
RF	Random forest
R_g_	Radius of gyration
RMSD	Root mean square deviation
RMSF	Root mean square fluctuation
SARs	Structure–activity relationships
SASA	Solvent-accessible surface area
SE	Sensitivity
SP	Specificity
SVM	Support vector machine
VDWAALS	Van der Waals interactions
