# Supplementary material for: Screening and Validation: AI-Aided Discovery of Dipeptidyl Peptidase-4 Inhibitory Peptides from Hydrolyzed Rice Proteins
Source: Foods. 2025 May 28;14(11):1916. doi: 10.3390/foods14111916 (PMC12154031; doi:10.3390/foods14111916)
Supplement: Supplementary file 1 [file foods-14-01916-s001.zip › foods-3589421-supplementary.pdf]

Supporting Information

Screening and Validation: AI-Aided Discovery of Dipeptidyl Peptidase-4 Inhibitory Peptides from Hydrolyzed Rice Proteins

*Cheng Cheng, Huizi Cui, Xiangyu Yu, Wannan Li **

Key Laboratory for Molecular Enzymology and Engineering of Ministry of Education, School of Life Sciences, Jilin University, Changchun, 130012, China

** Corresponding author.*

E-mail addresses: liwannan@jlu.edu.cn (W. Li).

Table S1. Oryza sativa glutelin and prolamin data from NCBI

NO.	Protein type	Protein ID	Protein Annotation
1	Oryza sativa Japonica Group	CAA29507.1	glutelin, partial
2	Oryza sativa Japonica Group	ABL74552.1	glutelin
3	Oryza sativa Japonica Group	ACT31355.1	glutelin, partial
4	\		glutelin
5	Oryza sativa	KAB8090568.1	hypothetical protein EE612_015772
6	Oryza sativa	KAB8087153.1	hypothetical protein EE612_011104
7	Oryza sativa	KAB8086667.1	hypothetical protein EE612_010114
8	Oryza sativa	KAB8083624.1	hypothetical protein EE612_005886, partial
9	Oryza sativa	KAB8082662.1	hypothetical protein EE612_004636
10	Oryza sativa	KAB8082442.1	hypothetical protein EE612_004322
11	Oryza sativa	KAB8082169.1	hypothetical protein EE612_003960
12	Oryza sativa	BAA92322.1	protein disulfide isomerase, partial
13	Oryza sativa Japonica Group	XP_066164194.1	glutelin type-B 1-like
14	Oryza sativa Japonica Group	XP_015624148.1	glutelin type-B 1-like
15	\	Q0ILQ6.1	Protein glutelin precursor
16	Oryza sativa Japonica Group	NP_001410566.1	ras-related protein Rab5A
17	Oryza sativa Japonica Group	BAF29772.1	Os12g0472500
18	Oryza sativa Japonica Group	BAF29598.1	Os12g0283400
19	Oryza sativa Japonica Group	BAF25378.1	Os09g0476800
20	Oryza sativa Japonica Group	BAF23982.1	Os08g0482300
21	Oryza sativa Japonica Group	BAH90862.1	Os01g0111900
22	Oryza sativa Japonica Group	BAF11132.1	Os03g0188500
23	Oryza sativa Japonica Group	BAF05612.1	Os01g0644600
24	Oryza sativa Japonica Group	BAF05442.1	Os01g0607000
25	Oryza sativa Japonica Group	BAT17103.1	Os12g0472500
26	Oryza sativa Japonica Group	BAS82698.1	Os03g0188500
27	Oryza sativa Japonica Group	BAS78514.1	Os02g0453600
28	Oryza sativa Japonica Group	BAS74462.1	Os01g0761800, partial
29	Oryza sativa Japonica Group	BAS73393.1	Os01g0644600
30	Oryza sativa Japonica Group	BAS73078.1	Os01g0607000
31	Oryza sativa Japonica Group	BAS70025.1	Os01g0111900
32	Oryza sativa Japonica Group	BAG98842.1	unnamed protein product
33	Oryza sativa Japonica Group	BAG98429.1	unnamed protein product
34	Oryza sativa Japonica Group	BAG98159.1	unnamed protein product
35	Oryza sativa Japonica Group	BAG97108.1	unnamed protein product
36	Oryza sativa Japonica Group	BAG96020.1	unnamed protein product
37	Oryza sativa Japonica Group	BAA11130.1	prolamin
38	Oryza sativa Japonica Group	BAA11129.1	prolamin

Table S2. Expasy——Peptide Cutter Simulated Hydrolysis Results

Enzyme Type	Main Recognition Site in Protein Sequence （from Expasy）	Number of Peptides	Total Number of Peptides
Trypsin	K*、R*	236	
Pepsin(PH>2)	*F、*Y、*W	394	
Proteinase K	A、E、F、I、L、T、V	478	
Chymotrypsin-high specificity (C-term to [FYW], not before P)	F*、Y*、W*	290	638
Chymotrypsin-high specificity (C-term to [FYW], not before P)+Proteinase K	F*、Y*、W*、A、E、F、I、L、T、V	473	
Trypsin+ Pepsin(PH>2)	K*、R*、*F、*Y、*W	513	

Note: Enzyme Type represents the enzymes used to simulate hydrolysis, including single and mixed enzymes; Recognition Site in protein sequence（from Expasy）denotes the amino acid site of cleavage，X*means the enzyme cuts at C-terminal respectively,*X means the enzyme cuts at N-terminal respectively,X means no specific direction; Number of peptides indicates the number of peptides hydrolyzed by each enzyme; Total number of peptides refers to the total number of peptides after removal of the same peptide.

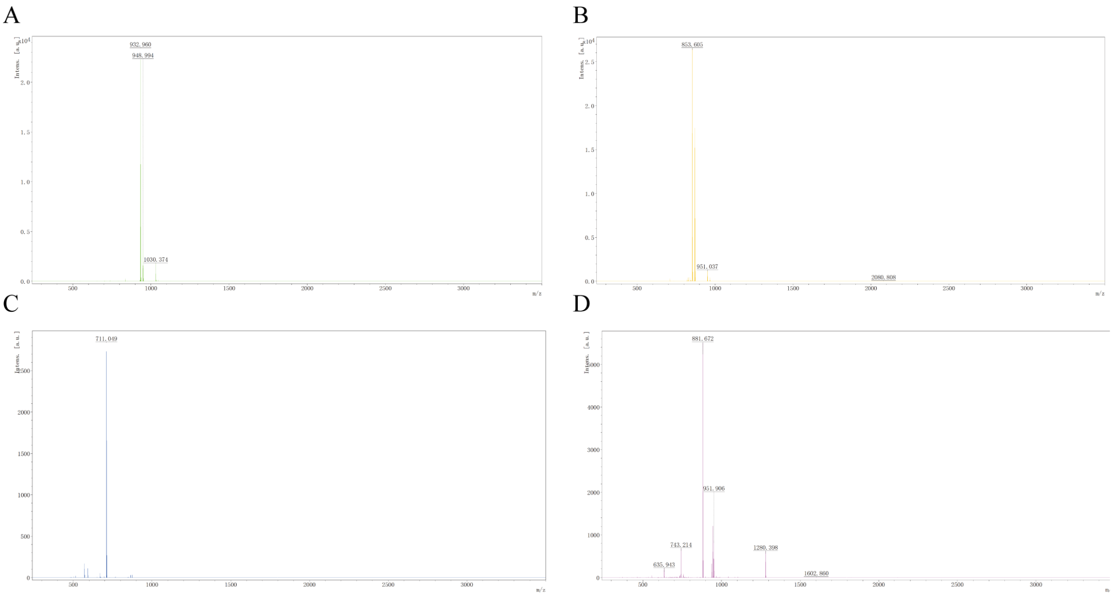

Figure S1. MALDI-TOF-MS of four synthetic peptides. PPPPPPPPA (A), PPPSPPPV (B), PPPPPY (C), and CPPPPAAY (D).
